# Growth hormone – releasing hormone in the immune system

**DOI:** 10.1007/s11154-024-09913-w

**Published:** 2024-10-07

**Authors:** Agnieszka Siejka, Hanna Lawnicka, Saikat Fakir, Nektarios Barabutis

**Affiliations:** 1 Department of Clinical Endocrinology, Medical University of Lodz, Lodz, Poland; 2 Department of Immunoendocrinology, Medical University of Lodz, Lodz, Poland; 3 School of Basic Pharmaceutical and Toxicological Sciences, College of Pharmacy, University of Louisiana Monroe, Monroe, LA 71201 USA

**Keywords:** GHRH, GHRH antagonist, Immune system, Inflammation, Endothelium

## Abstract

GHRH is a neuropeptide associated with a diverse variety of activities in human physiology and immune responses. The present study reviews the latest information on the involvement of GHRH in the immune system and inflammation, suggesting that GHRH antagonists may deliver a new therapeutic possibility in disorders related to immune system dysfunction and inflammation.

## Introduction

GHRH physiologically synthesized in the hypothalamus, was first isolated from a pancreatic neuroendocrine tumor causing acromegaly [1]. GHRH increases the secretion of GH from the anterior pituitary gland. The extrapituitary activities of GHRH are the focus of intense investigations [2–4]. The paracrine and autocrine actions of GHRH lead to increased cancer cell proliferation and spread, while GHRHAnt exert opposite effects [4, 5]. Effects of GHRH in the immune system and inflammation will be summarized below.

### GHRH

GHRH is a 44 amino acid peptide synthesized in the hypothalamic arcuate nucleus, but its full biological activity is retained in the first 29 amino acids. In humans both 44- and 40- amino acid forms are present. GHRH acts on the anterior pituitary gland, where it stimulates the synthesis and release of GH. The downstream target of GHRH/GH axis is the IGF-1 produced in the liver, which is the mediator of GH actions in peripheral tissues. The half-life of 1–40 GHRH is 3.9 min [6] and it is inactivated by peptidases.

### pGHRH-R

The pGHRH-R is a member of a seven-transmembrane class B G-protein coupled receptor family, which also includes the receptors for vasoactive intestinal peptide, pituitary adenylyl cyclase-activating peptide, secretin, glucagon, glucagon-like peptides, calcitonin, and gastric inhibitory polypeptide [7]. The binding of GHRH to GHRH-R activates second messengers which include adenylate cyclase–cAMP–PKA and Ca^2+^-calmodulin, inositol phosphate–diacylglycerol–protein kinase C (PKC), L-type calcium channels, and arachidonic acid–eicosanoic pathways. Activation of pGHRH-R ultimately results in the stimulation of GH synthesis and secretion [8].

### Splice variants (SVs) of GHRH-R

SVs of the pituitary type GHRH-R were first isolated form cancer cells [9]. The SV1 of GHRH-R differs from the pGHRH-R in the N-terminal extracellular domain. The first 89 aminoacids of the pGHRH-R are replaced in the SV1 receptor by a different 25-amino acid sequence. SV1 receptor possesses ligand-dependent and ligand-independent activities [10]. The binding of GHRH to SV1 receptor leads to MAPK activation and increased cell proliferation [11].

### Neuroendocrine-immune communication

The nervous, endocrine and immune systems cross-talking is well established [12, 13] and utilizes hormones and receptors—widely expressed in the immune cells—to maintain body homeostasis [12, 14]. Cytokines also modulate hormone synthesis and release from the endocrine glands and affect central nervous system function [12, 14]. High concentration of cytokines during acute or chronic illness decreases hormones secretion and leads to hormone resistance, impairing endocrine tissue function [12, 15, 16]. This may be considered a self-defense approach of the body to limit energy expenditure to combat disease [14]. Cytokine-induced hormone resistance has implications in a number of disorders and disease states ranging from diabetes and other autoimmune diseases to clinical depression [13, 14].

### GHRH and GHRH receptors in immune cells and neurons

Immunoreactive GHRH, mRNA encoding for GHRH, and GHRH binding to rodent lymphocytes was first reported in 1990 [17]. Shortly after, it was demonstrated that human lymphocytes synthesize and release biologically active GHRH, able to stimulate GH release from the pituitary and lymphocytes [18]. Later on, it was revealed that GHRH receptors are present on peripheral blood mononuclear cells (PBMC) [19]. GHRH was expressed in less than 2% of human PBMC [20]. PBMCs and granulocytes express low levels of GHRH mRNA with relatively higher levels of expression in monocytes [20]. A recent study which utilized Western blot and immunofluorescence revealed the presence of GHRH receptors and its splice variant SV1 in both THP-1 cells and PBMCs [21]. GHRH exists is monocytes, B and T cells, but its expression in T cells is 5–7 fold higher than in B cells and monocytes.

GHRH expression is also dependent on age and gender. GHRH mRNA expression in PBMC derived from postmenopausal women is lower than that of premenopausal women [20]. PBMCs from women receiving HRT secrete more GHRH *in vitro* than cells from women which do not receive hormone replacement [20]. GHRH can also affect the release of several cytokines from PBMC. GHRH significantly increased the concentration of IFN-γ, intereukin 17 (IL-17), IL-2 and sIL-2Rα in the supernatants of cultured PBMC *in vitro* [22–24]. It was further confirmed that GHRH acts as a potent stimulator of cytokine synthesis and release [5]. GHRH-R is not expressed in naïve CD4^+^ T cells, while its expression is induced throughout Th17 cell differentiation *in vitro* [25].

GHRH-R activates the JAK-STAT3 [26] and NF-κB [27] pathways, which play crucial role in immune responses to various stimuli. In a rat model of endotoxin-induced uveitis, LPS induced the expression of GHRH-R; which activated the JAK2/STAT3 pathway and increased the production of IL-6, IL-17A, COX2, and iNOS. MIA-602—a GHRHAnt- inhibited JAK2/STAT3 activation [28]. Lipopolisaccharide (LPS) triggered NF-κB activation increased the expression of inflammatory and pro-oxidative markers in mouse prefrontal cortex, while GHRHAnt exerted anti-inflammatory and antioxidative effects.

GHRHAnt demonstrated anxiolytic and antidepressant effects in the brain [29]. *In vivo*, GHRH delivered by plasmid injection and electroporation led to increased numbers of CD2 + αβ T-cells, CD25 + CD4 + cells, and CD4 + CD45R + cells compared to controls. At 300 days post-GHRH therapy, CD45R + /CD45R0 − naïve lymphocytes were significantly increased, as well as the Natural killer lymphocytes (CD3 − CD2 +); resulting to better body condition scores [30].

In humans, a 16-week administration of [norleucine^27^] GHRH (1–29)-NH_2_ at a dose of 10 μg/kg once daily led to a significant (30%) increase in B cells (CD20), 20% increase in cells expressing the T cell receptor α/β, and 40% increase in cells expressing T cell receptor γ/δ. There were no changes in the number of T cells (CD3), T cell subsets (CD4, CD8), or natural killer cell (CD57) during treatment. There was also an increase in B cell numbers associated with enhanced responsiveness (50%) to B cell mitogens, as well as an increase—at 4 weeks—in circulating IgG, IgM, and IgA. T cells responsiveness to phytohemagglutinin was elevated by 50%. Moreover, there was also a 70% increase in the number of lymphocytes expressing the IL-2R (CD25), and enhanced IL-2R messenger RNA expression and basal IL-2 secretion at 16 weeks of treatment. Circulating soluble IL-2 receptor rose significantly within 4 weeks of treatment and remained elevated for the duration of the study. There were no sex differences in the immune response to GHRH analog and no adverse effects [31]. GHRH-R is also expressed on mouse bone marrow—derived mesenchymal stem cells and drives GHRH agonist stimulation of STAT3 phosphorylation [32]. Individuals with severe IGHD caused by the *null* homozygous (c.57 + 1 A → G) mutation in the GHRH receptor gene in Northeast Brazil are characterised by relatively reduced spleen volume and total serum IgG levels. Moreover, they present with smaller papule diameter after streptokinase injection, although they do not exhibit increased frequency of infections [33]. The aforementioned observations suggest that GHRH possesses immunomodulatory properties [34].

GHRH stimulates the synthesis and secretion of GH from the pituitary. In the elderly GH levels are decreased and the state is called somatopause. This is related to decreased GHRH content in the hypothalamic neurons [35]. The number of GHRH neurons in the hypothalamus decreases with age [35, 36], hence the synthesis of this neurohormone becomes lower and the GHRH-stimulated GH synthesis and immune response is affected, making those individuals more susceptible to chronic diseases and shorten health span [37]. GHRH receptors are expressed on AML and promyelocytic leukemia cells, and the GHRHAnt MIA 602 significantly inhibits proliferation of leukemia cell lines *in vitro* [38, 39].

### GHRH in autoimmunity and inflammation

GHRH-R is a mediator of the GHRH-stimulated synthesis of GH, which further increases IGF-1 levels. It was reported that GHRH is crucial for the development of EAE [40]. The authors demonstrated that GHRH-deficient *lit/lit* mice do not develop EAE [40], and that this condition was alleviated in both GHRH-KO and GHRHR-KO mice [41]. GH supplementation was able to restore EAE susceptibility in the GHRH-KO mice, suggesting that GH—but not GHRH—is involved in the development of EAE [41]. IGF1 has also been reported to affect the development of EAE in mice [42]. Altogether, the above studies show indirect effects of GHRH on EAE development.

LPS induced GHRH-R and SV1 expression in infiltrating immune cells in the iris and ciliary body of the eye [43]. GHRHAnt diminished LPS-induced production of TNF-α, IL-1β, and MCP-1 [43]. The mRNA levels of genes associated with pathogenic Th17 cells, including *Il17a, Il17f, Il22*, and *Csf2* were decreased, while *Il10* was increased, in the CD4^+^ T cells isolated from the eyes of *Ghrhr*^*lit/lit*^ mice [25]. In human ciliary epithelial cells, the NF-κB subunit p65 was phosphorylated in response to stimulation with LPS, resulting in transcriptional up-regulation of GHRH-R [28].

It has been also demonstrated that GHRHAnt inhibited proliferation of prostatic epithelial cells induced by chronic inflammation, and thus alleviated autoimmune prostatitis [44]. Moreover, those peptides decreased colon and lung inflammation [45, 46] and inhibited inflammation in sarcoidosis [47]. Inflammation and cancer coexist [3, 48], and cytokines/chemokines facilitate carcinogenesis [49]. Immunomodulatory therapeutic approaches are successfully applied in oncological treatment [49].

Chronic inflammation may also lead to excessive production of reactive oxygen and nitrogen species, which alter immune responses and lead to oncogenic transformations [3, 50]. The innate immune system depends on ROS to maintain human tissue integrity and combat pathogens, and are generated by mitochondria due to activation of several proinflammatory pathways (e.g. MAPK, AMPK, PI3K/ACT) in coordination with NFκB and HIF1α [51]. GHRH stimulates these pathways and ROS production while GHRHAnt counteract those events [52–54].

MIA602 modulated lung inflammation and inhibited fibrosis due to bleomycin. Those effects were mediated by modulation of T-cell signaling and reducing inflammation [46]. Mice lacking GHRH gene are smaller than normal controls, with lower thymocyte number and B lymphopenia. Mutant mice show a constant increase in CD4 T cells and decrease in CD8 T-cell frequency compared to normal mice [55]. Mice lacking GHRH (*Ghrh*^−/−^ mice) exhibited high susceptibility to *S. pneumoniae* infection with a time-dependent increase in lung bacterial load and a lethal bacteraemia after 24 h. Lungs of infected *GHRH*^−/−^ mice were massively infiltrated by inflammatory macrophages and neutrophils, while lung B cells were markedly decreased. In contrast wild type animals completely cleared bacteria after 24 h [56]. *GHRH*^−/−^ mice were unable to trigger production of specific IgM and IgG against serotype 1 pneumococcal polysaccharide (PPS) after vaccination with either native PPS (Pnx23) or protein-PPS conjugate (Prev-13) vaccines [57].

### GHRH-related analogs in endothelial inflammation and sepsis

The endothelial cells are crucial modulators of tissue function and survival, and their dysregulation can present as either the cause—or the consequence—of severe tissue (e.g. lung, brain) impairment. They form semipermeable monolayers which line blood vessels, in order to regulate gas and nutrient exchange between the blood and the underlying tissues. Endothelial permeability depends on many factors, including the interconnected network of cytoskeletal proteins and adherent junctions [58]. Elucidation of the cellular cascades involved in barrier integrity and compromise is an active area of investigation of many disciplines. The development of targeted therapeutics to ameliorate excessive endothelial leak contributes in the development of efficient medical interventions towards devastating diseases; including sepsis, acute respiratory distress syndrome, and neurological disorders [5, 59, 60].

As previously mentioned, GHRH exerts a growth factor activity in prostate, breast and lung cancer cells [61, 62]. GHRHAnt were developed to treat malignancies, and have been associated with anti-inflammatory and anti-oxidative activities in lung, brain endothelial cells and mouse lungs [21, 63–72]. Moreover, they ameliorate injury in septic lungs [53] and toxin-induced endothelial injury [73]. The targeted actions of GHRHAnt via GHRH receptors set them apart from other anti-inflammatory agents, such as Hsp90 inhibitors [74–82].

GHRHAnt induce P53 [3, 83–85]. P53 is a tumor suppressor protein associated with robust anti-inflammatory activities in the endothelium [78, 80, 86–91], which supports barrier function via Rac1/RhoA regulation [80, 87]. P53 omission in mice exacerbate inflammatory responses [90, 92]; whereas super-P53 mice—which globally overexpress P53—are protected against LPS-induced lung injury [87, 93]. Interestingly, P53 is phosphorylated and reduced by bacterial toxins [78, 80, 88, 94], and UPR can modulate that modification [95, 96]. The exacts kinases involved in those effects are not known, but their crucial role in endothelial permeability has been reported before (reviewed in [97]). It is possible that NEK kinases may be involved in those events, since it was previously shown to affect P53 ( reviewed in [98]), and to be induced in septic mice [99, 100]. Further studies revealed that NEK2 and NEK9 inhibition suppresses LPS-induced endothelial leak [64].

GHRHAnt induce UPR [101], and UPR suppression opposes the beneficial effects of those peptides in the inflamed endothelium [101, 102]. This is important because of the major role of UPR in barrier regulation and inflammation. In particular, UPR suppression due to kifunensine induces hyperpermeability [103, 104] whereas Brefeldin A and kifunensine – which induce UPR—modulate LPS-induced endothelial leak in human and bovine cells [105]. Moreover, the UPR suppressor 4-Phenylbutyrate potentiates LPS-induced endothelial injury [106, 107]; and Tunicamycin – a UPR inducer – protects against inflammation since it reduces paracellular and transendothelial hyperpermeability due to LPS, ameliorates cytoskeletal remodeling and inflammation; and reduces the internalization of VE-cadherin, enhancing endothelial integrity [108, 109].

GHRHAnt induce the UPR sensor ATF6, previously involved in disease protection (reviewed in [110–112]) and barrier regulation. Targeted ATF6 suppression due to Ceapin-A7 or small interfering RNA potentiated LPS-induced endothelial breakdown. AA147 – induced ATF6 activation prevented LPS-induced barrier disruption by counteracting cofilin and MLC2 activation, as well as VE-Cadherin phosphorylation [113–115]. Furthermore, Ceapin–A7 potentiated inflammation [116, 117]. GHRHAnt induce both PERK and IRE1α, but information on their role in endothelial permeability and inflammation is very limited. Western blot analysis of phosphorylated IRE1α and IRE1α in mouse lungs treated with either vehicle (saline) or LPS (1.6 mg/kg) via an intratracheal injection for 24 h, revealed that LPS reduces IRE1α levels in the inflamed lungs [118]. Indeed, UPR regulates P53 expression in the pulmonary endothelium. Brefeldin A, dithiothreitol, and thapsigargin induced P53 expression levels. When the cells were treated with N-acetyl cysteine, kifunensine, and ATP-competitive IRE1α kinase-inhibiting RNase attenuator, P53 was reduced [119]. Both P53 and UPR are targeted by GHRHAnt. The aforementioned observations might have shed light onto the pathways involved in endothelial permeability and inflammation regulation, but many questions remained unanswered, including the effects of PERK, IRE1α and P53 in endothelial leak and inflammation as it relates to the effects of GHRH analogs towards key cytoskeletal proteins; as well as the involvement of NEKs [120].

Recent investigations have focused on the role of GHRHAnt in endotoxin-induced endothelial damage. *In vitro*, the JV-1–36 antagonist ameliorated barrier dysfunction and reduced ROS generation due to LPS or LTA treatment in HUVECs. NEK2 expression levels were increased in the inflamed cells, and JV-1–36 counteracted those endothelial events, supporting the beneficial effects of GHRHAnt in toxin-induced endothelial injury [73]. NEK2 exaggerates and potentiates inflammatory responses, and degrades P53. Interestingly, GHRHAnt ameliorated IFN-γ—induced paracellular hyperpermeability and reactive oxygen species generation in bovine and human pulmonary endothelial cells; and suppressed the corresponding STAT3, cofilin and ERK1/2 activation [68].

**Fig. 1:**
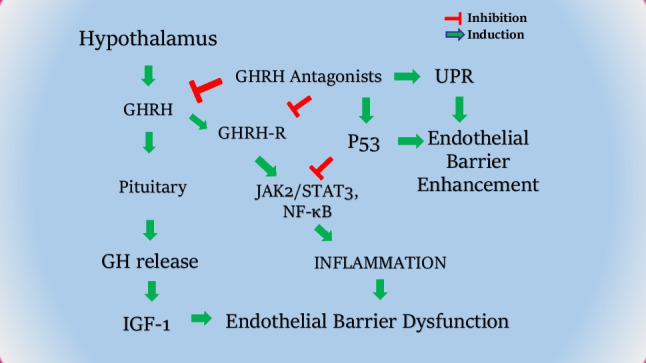
GHRH involvement in endothelial barrier and inflammation regulation. GHRH regulates the secretion of GH from the anterior pituitary gland, to induce IGF-1 and inflammatory cascades. GHRHAnt are able to counteract GHRH-induced pathways (e.g. JAK/STAT3) and activate UPR; to ameliorate barrier dysfunction, a condition related to lung injury

It was recently revealed that GHRHAnt exert beneficial effects in the septic lungs of mice subjected to cecal ligation and puncture – induced sepsis. JV-1–36 significantly suppressed IL-1α, IL-6, and pSTAT3 activation in the septic lungs. Moreover, GHRHAnt treatment reduced bronchoalveolar lavage fluid (BALF) protein concentration, in line with previous results in a model of acute lung injury [69]. The above-mentioned observations on endothelial barrier function and inflammation are summarized in Fig. 1.

### GHRH, GHRHAnt and COVID-19

COVID-19 pandemic led to increased mortality due to acute respiratory distress syndrome (ARDS) [121] but also enabled a better understanding of underlying mechanisms of host defense and virus-induced autoimmunity. It is known that viruses replicate and activate the host immune response, which may lead to the development of local or systemic inflammation. Those events result in the destruction of infected tissues and dysfunction of multiple organs, including those of the endocrine system; as it was demonstrated in the thyroid, adrenals and pituitary during COVID-19 [122–127]. SARS-CoV-2 spike (S) protein enables the virus to enter the cell and promotes inflammatory responses, including production of inflammatory cytokines/chemokines, cytokine storm and lung inflammation [128].

SARS-CoV-2 infection induced cytokine storm and led to acute lung injury (ALI) and ARDS. MIA-602 (GHRHAnt) reduced inflammation induced by SARS-CoV-2 spike protein in THP-1-derived macrophages, inhibited NF-κB, JAK-STAT and MAPK inflammatory pathways, reduced ROS production, inhibited COX-2 and iNOS expression and attenuated NF-κB activity in PBMCs [21]. MIA-602 also acted anti-inflammatory in SARS-CoV-2 infected mice *in vivo* [121]. The same antagonist reduced lung perivascular inflammation/pneumonia, decreased lung/heart ICAM-1 expression and normalized airflow parameters [121]. Individuals with iGHD were shown to cope better with the SARS-CoV-2 infection than controls; this might be related to altered secretion of adipokines and cytokines like interleukin 6 (IL-6), TNF-α and interferon, which altogether may limit immune-related tissue destruction due to the infection with SARS-CoV-2 [129].

## Conclusions

In our view, the role of GHRH and its synthetic analogs in the field of immunoneuroendocrinology and endothelial biology is prominent (Table 1). Further efforts in experimental models of disease and clinical trials will reveal the wide spectrum of its involvement in human pathophysiology and pharmacotherapy.

**Table 1:** GHRH functions

Topics	Description	Function/Role	References
GHRH	• 44-amino acid peptide synthesized in the hypothalamus • Exerts extra-pituitary effects, increases cancer proliferation	• Stimulates GH secretion from the pituitary	[6, 20]
pGHRH-R	• G-protein-coupled receptor	• Regulates GH synthesis and secretion	[8]
SV1	• SV1 differs from the pGHRH-R in the N-terminal extracellular domain	• Promotes cancer cell proliferation and ligand-dependent/independent activities	[10, 11]
Neuroendocrine-immune crosstalk	• Interaction between the nervous, endocrine, and immune system	• Maintains homeostasis, modulates hormone synthesis, and immune function	[12–14]
GHRH in PBMCs and monocytes	• GHRH mRNA expression is present in peripheral blood mononuclear cells (PBMCs) and granulocytes, with higher expression levels in monocytes	• Affects the release of several cytokines (e.g. IFN-γ, IL-17, and IL-2) enhancing immune responses	[19, 20, 22]
GHRH-R signaling	• Modulates cytokine production, inflammation, and immune cell differentiation	• GHRH-R activates JAK-STAT3 and NF-κB to modulate immune responses	[26–28]
GHRH and aging	• The number of GHRH neurons in the hypothalamus decreases with age, leading to reduced GH synthesis and immune response	• Reduced GHRH content in elderly individuals is associated with increased susceptibility to chronic diseases	[35, 37]
GHRH in endothelial cells	• GHRH modulate inflammation, oxidative stress, and endothelial integrity	• GHRHAnt suppress LPS-induced lung injury and support endothelial barrier function	[53, 63, 64]
GHRH in autoimmunity	• Crucial for the development of EAE, an experimental model of multiple sclerosis • GHRH-KO mice show resistance to EAE, suggesting GH involvement in EAE development	• GHRH indirectly affects the development of EAE through GH and IGF-1 • Deficiency or receptor knockout alleviates EAE, while GH supplementation restores susceptibility	[40–42]
GHRH in cancer and inflammation	• GHRH stimulates pro-inflammatory pathways (e.g. MAPK, AMPK, and PI3K/AKT) to increase the production of reactive oxygen species	• Facilitates cancer development by influencing inflammation and oxidative stress pathways	[3, 50, 51]
GHRHAnt	• Exert anti-inflammatory and anti-oxidative effects in endothelial and lung tissues • Decrease the synthesis of hepatic IGF-I	• Block GHRH-R activity • Protect against endotoxin-induced damage and decrease ROS production • Inhibit cancer cell proliferation	[28, 29, 72]
GHRHAnt, UPR and P53	• GHRHAnt induce UPR to regulate endothelial permeability and inflammation	• GHRHAnt induce unfolded protein response (UPR) and activate tumor suppressor protein P53 • UPR and P53 modulate barrier integrity and reduce inflammation	[101, 103, 113]
GHRHAnt in sepsis and lung Injury	• Influence lung inflammation, fibrosis, and sepsis through the modulation of T-cell signaling, cytokine production, and oxidative stress	• Reduce lung damage and inflammation in septic conditions	[46, 53, 118]
GHRHAnt in NEK2	• NEK2 is upregulated in inflammatory conditions and involved in P53 degradation • Potentiates inflammation and endothelial leak in response to toxins	• GHRHAnt suppress NEK2, protecting against inflammation-induced damage	[68, 73, 120]

## Data Availability

No datasets were generated or analysed during the current study.

## Abbreviations

GH: Growth hormone
GHRH: Growth hormone-releasing hormone
GHRHAnt: Growth hormone-releasing hormone antagonist
IGF-1: Insulin-like growth factor 1
GHRH-R: Growth hormone-releasing hormone receptor
pGHRH-R: Pituitary-type GHRH receptor
cAMP: Cyclic adenosine monophosphate
PKA: Protein kinase A
SVs: Splice variants
MAPK: Mitogen-activated protein kinase
mRNA: Messenger ribonucleic acid
THP-1: Tohoku hospital pediatrics-1
PBMC: Peripheral blood mononuclear cell
IFN-γ: Interferon gamma
sIL-2Rα: Soluble IL-2 receptor alpha
CD: Cluster of differentiation
NF-κB: Nuclear factor kappa B
JAK: Janus kinase
STAT3: Signal transducer and activator of transcription 3
COX2: Cyclooxygenase 2
IL-2R: Interleukin-2 receptor
IGHD: Isolated GH deficiency
AML: Acute myeloid leukemia
EAE: Experimental autoimmune encephalomyelitis
KO: Knock out
TNF-α: Tumor necrosis factor alpha
MCP-1: Monocyte chemotactic protein-1
ROS: Reactive oxygen species
AMPK: Adenosine monophosphate-activated protein kinase
PI3K: Phosphoinositide 3-kinase
HIF1α: Hypoxia-inducible factor 1 subunit alpha
Hsp90: Heat shock protein 90
UPR: Unfolded protein response
NEK: Never in mitosis A (NIMA)-related kinase
ATF6: Activating transcription factor 6
MLC2: Myosin light chain 2
PERK: Protein kinase R-like ER Kinase
IRE-1α: Inositol-requiring enzyme 1 alpha
ATP: Adenosine triphosphate
LPS: Lipopolysaccharide
LTA: Lipoteichoic acid
HUVEC: Human umbilical vein endothelial cells
ICAM-1: Intercellular adhesion molecule-1
ALI: Acute lung injury
ARDS: Acute respiratory distress syndrome
